# VICINO@TE, distant but together, new app to communicate with families living in complete isolation during COVID-19 pandemic

**DOI:** 10.1186/s13054-020-03319-1

**Published:** 2020-11-27

**Authors:** Giovanni Pedrotti, Angelo Attilio Colombo, Fabrizio Corradini, Rosella Martini, Massimiliano Raggi, Mariavittoria Modena

**Affiliations:** 1 Anesthesia and Intensive Care, APSS, Rovereto Hospital, Rovereto, Italy; 2 Intensive Care, APSS, Rovereto Hospital, Rovereto, Italy

ICU admission of a relative is a stressful event for family members, something that may lead to high levels of distress throughout their relative’s hospitalization and cause symptoms of post-traumatic stress disorder and other psychological symptoms after the ICU [1].

Good communication between ICU staff and patients’ relatives is generally accepted to be a preventive factor of such problems, not only for patients’ physical and psychological health but also for the health of the patients’ relatives. It is the responsibility of healthcare professionals to ensure that patients’ relatives are informed about what happens during and after an ICU stay [2].

The outbreak of coronavirus disease 2019 (COVID-19) [3] has created a global health crisis that has had a deep impact on the way we communicate with patients and their relatives in all the COVID-19 care settings, given the need to maintain isolation and social distancing [3, 4].

For this purpose, the SIAARTI (Italian Society of Anesthesia, Analgesia, Intensive Care) has released a guideline on how to communicate with families in isolation, which advises how to set up phone calls [5]: Family members must be given clinical information at least once a day, and more often in case of any substantial and unexpected deterioration in the patient’s condition. These daily communications must cover the diagnosis and prognosis. Information can be provided by any means (e.g., telephone, video call, or e-mail).

VICINO@TE is an Internet portal/app that makes it possible for family members to receive updates or request clinical information regarding their loved one, to see pictures or videos of them, and to send them messages full of support and love.

Using a web/app platform, a virtual ward has been created where each patient is assigned a bed. An e-mail requesting access to the service is sent to the contact person for the family member, which after its compilation, allows to identify and guarantee the adequate confidentiality of the information and the collection of the necessary consents. After these preliminary operations, the family member receives the access credentials to the platform which allow them to access their loved one’s information in complete safety. Subsequently, each patient can be associated with a space where multimedia content or written texts can be inserted by staff (photos or medical bulletins) or relatives (texts, photos, videos) as shown in Fig. 1.

**Fig. 1:**
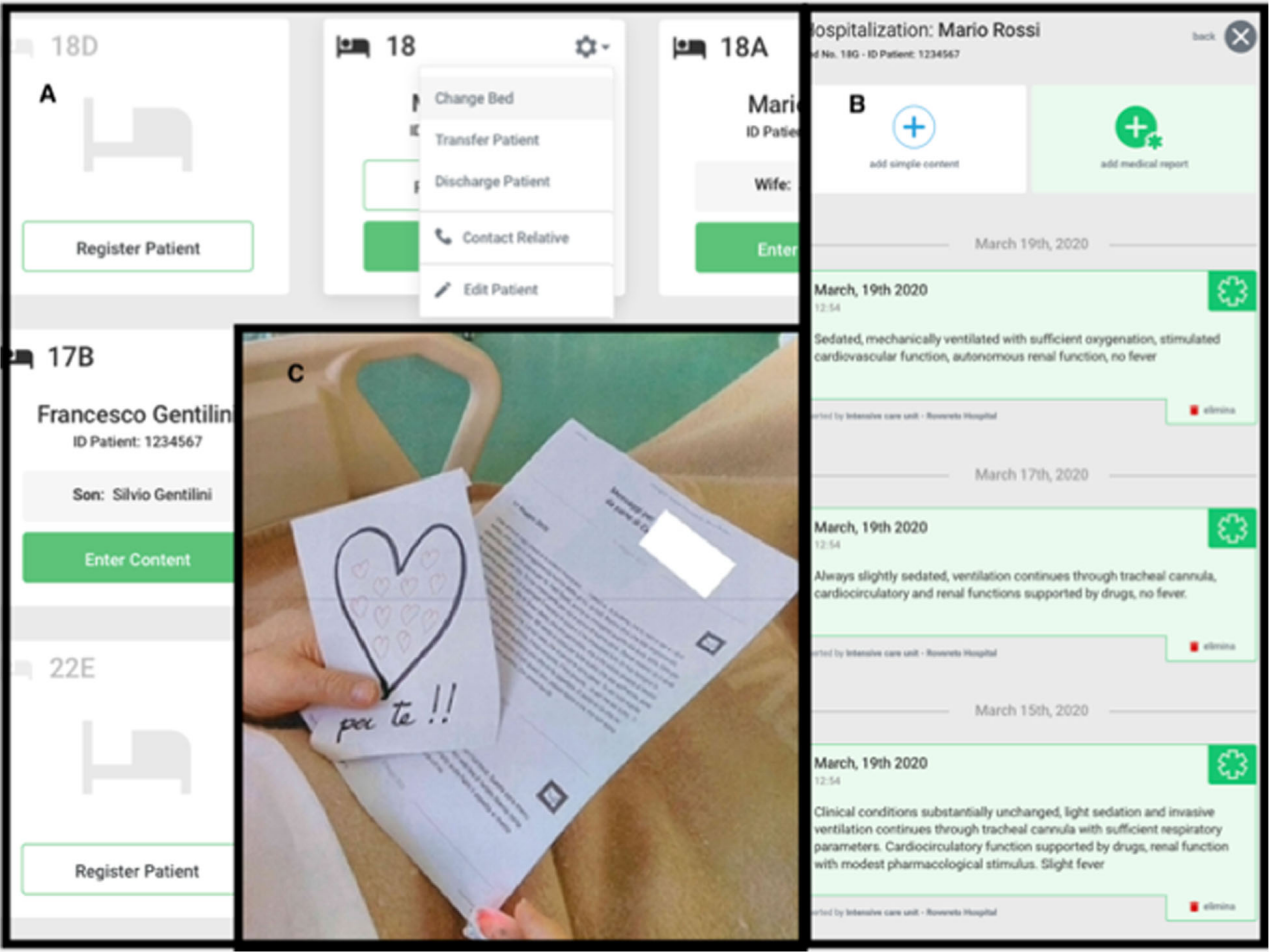
Screenshot of the app. **a** “Virtual” beds (examples) in which can be deposited written information (medical reports) and multimedia content, which can be either from the hospital workers or from families. **b** Example of a daily medical report. **c** The patient receives a printed copy of the letter that the family has written and deposited on the virtual bed

The service was made possible thanks to the availability of an IT company in Trentino and with the coordination by the Digital Administration Policies service. It is owned by the APSS which, upon request, can quickly grant it for free reuse or provided as a service to others healthcare companies.

Once the epidemic was over, we interviewed relatives of the patients by telephone regarding the satisfaction of the application. Out of the 63 patients hospitalized for COVID-19, it was possible to implement the application to 58 of them. Forty-six patients replied to the interview and 45 said they were satisfied, and 31 people appreciated the multimedia content (photos and videos) as well as the medical bulletins, but 14 underlined their preference for calls only.

## Data Availability

None

## References

[1] Davidson JE, Jones C, Bienvenu OJ. Family response to critical illness: postintensive care syndrome-family. Crit Care Med. 2012. 10.1097/CCM.0b013e318236ebf9 22080636

[2] Davidson JE, Aslakson RA, Long AC, Puntillo KA, Kross EK, Hart J, et al. Guidelines for family-centered care in the neonatal, pediatric, and adult ICU. Crit Care Med. 2017. 10.1097/CCM.0000000000002169 27984278

[3] Nacoti M, Ciocca A, Giupponi A, Brambillasca P, Lussana F, Pisano M (2020). At the epicenter of the Covid-19 pandemic and humanitarian crises in Italy: changing perspectives on preparation and mitigation.

[4] Marra A, Buonanno P, Vargas M, Iacovazzo C, Ely EW, Servillo G. How COVID-19 pandemic changed our communication with families: losing nonverbal cues. Crit Care. 2020. 10.1186/s13054-020-03035-w PMC7274511 32503605

[5] COMMUNICoViD position paper: how to communicate with families living in complete isolation joint document SIAARTI, Aniarti, SICP, SIMEU. Version 01 Published on April 18, 2020 available at https://www.simeu.it/w/download/get/0/CommuniCoViD_eng%20-%2018apr20%20(003).pdf/download/articoli/4052.

